# Cholesterol Sulfate in Biological Membranes: A Biophysical Study in Cholesterol-Poor and Cholesterol-Rich Biomimetic Models

**DOI:** 10.3390/membranes15060159

**Published:** 2025-05-24

**Authors:** Ana Reis, Maria João Sarmento, Mariana Ferreira, Paula Gameiro, Victor de Freitas

**Affiliations:** 1 Rede de Quimica e Tecnologia—Laboratorio Associado de Quimica Verde, Departamento de Química e Bioquímica, Faculdade de Ciências, Universidade do Porto, Rua do Campo Alegre, 4169-007 Porto, Portugal; mariana.ferreira@fc.up.pt (M.F.); agsantos@fc.up.pt (P.G.); vfreitas@fc.up.pt (V.d.F.); 2 Instituto de Medicina Molecular, Faculdade de Medicina, Universidade de Lisboa, 1649-028 Lisboa, Portugal; maria.sarmento@gimm.pt

**Keywords:** plasma membrane, membrane fluidity, membrane order, surface charge, microbial cholesterol sulphate

## Abstract

As a surface-located molecule in biological membranes, cholesterol sulphate (CholS) plays a major role in membrane-driven cell–cell processes and events including platelet-cell adhesion, T-cell receptor signalling, sperm–egg interaction, membrane fusion, and skin differentiation. Despite this, little is known about the biophysical implications of CholS at the membrane in cells and organelles. In this study, we investigate the effect of increasing the content of CholS on the biophysical properties in cholesterol-poor and cholesterol-rich biomimetic models. Data obtained show that increasing amounts of CholS result in a slight increase in anisotropy, evidence for decreased membrane fluidity at higher CholS content (10 mol%) in cholesterol-poor systems but only negligible in rigidified epithelial-like cholesterol-rich systems. On the other hand, incorporation of CholS has an overall increasing ordering effect on membrane organisation and on-surface potential that is influenced by the lipid composition and cholesterol content. Though further research is needed to gain better insights on the (patho)physiological levels of CholS in cells and organelles, our findings are discussed in the context of diet–microbiota–host interactions in membrane-driven events in inflammatory-related disorders.

## 1. Introduction

Cholesterol sulphate (CholS), the sulfoconjugated form of cholesterol (Chol), belongs to the larger heterogeneous class of lipid sulphates [1,2]. Among lipid sulphates, CholS is one of the most important sterol sulphates and is widely distributed across body fluids, cells, and tissues [1,3]. Physiological levels of CholS are intimately related to its formation by endogenous sulfotransferase (SULT2B1) enzymes [4,5] and by gut microbiota with sulfotransferase activity [6], and their clearance by microsomal steroid sulfatases [5]. Even though information on CholS basal levels in circulation, cells, and tissues, and any changes introduced by diet, age, and disease remain scarce [1,2,3,7,8,9], plasma and tissue CholS levels were found significantly elevated in pathological conditions [8,10,11,12,13,14]. Possibly the most well-known condition relates to the scaling of the skin in patients with recessive X-linked ichthyosis (RXLI) disorder, characterised by a deficiency of steroid sulfatase (STS) resulting in an excess of CholS at the tissue level that can reach up to 12.5% of total lipids [7,8]. In addition, deregulated CholS levels have also been reported in colonic tissue of patients with ulcerative colitis [14] and in the prostate tissue of patients with high-grade intraepithelial neoplasia [11]. Nonetheless, to date, knowledge on the content of CholS in tissues and its distribution across cells and organelles remains largely unknown.

Despite the scarcity of information on the physiological levels of CholS in health and disease, it is generally accepted that CholS is mostly confined to specific regions [8], particularly within sphingolipid-cholesterol-rich domains [13,15] and thus becoming involved in membrane-driven cell–cell events including T-cell receptor signalling [16], platelet–cell adhesion [17], membrane fusion [18], sperm–egg interaction [13] and skin differentiation [19]. Recent investigations revealed that increased levels of CholS was able to modulate host immune cell trafficking, whereby limiting neutrophil recruitment and exacerbated inflammatory response in intestinal mucosa contributed to intestinal mucosa barrier healing [14,20], ameliorating inflammation in intestinal diseases.

Regardless of its significance in biological events, the biophysical relevance of CholS at the membrane level remains poorly understood. Due to its charged sulphate group, CholS is less embedded in the lipid bilayer, remaining at the water–lipid interface in biomembranes where it contributes to the decrease in the membrane’s surface potential [21,22] and the stabilisation of membranes, as reported in red blood cells [23]. Being located at the water–lipid interface, previous biophysical work conducted with artificial phosphatidylcholine (PC) membranes has shown that the presence of CholS induces a decrease in the lipid transition temperature [24] and a slight ordering effect [21,25]. Other researchers working with biomembranes mimicking the stratum corneum (SC), composed of ceramides:free fatty acids:Chol, found that increasing ratios of CholS, as typically reported in RXLI [7,8], fluidized the sterol component of the lipid mixture resulting in decreased lipid rigidity and increased permeability of these SC biomembranes, which was consistent with increased skin permeabilities in diseased phenotypes [22]. However, in their experimental design [22], the incorporation of CholS was accompanied by replacement of Chol, changing the Chol/lipid ratios and making it ambiguous as to whether the reported effects were related to the increasing amounts of CholS or the decrease in Chol levels. Also, most of the experimental and theoretical work focused on understanding the impact of CholS on the biophysical properties of membranes [21,24,25] was carried out in oversimplistic PC models, which are hardly a representation of biological membranes. Cell membranes and other organelles have characteristic lipid composition and distinct cholesterol-to-phospholipid (Chol/PL) ratios varying according to the species, cell type, organelle, and membrane leaflet [26,27].

**Figure 1:**
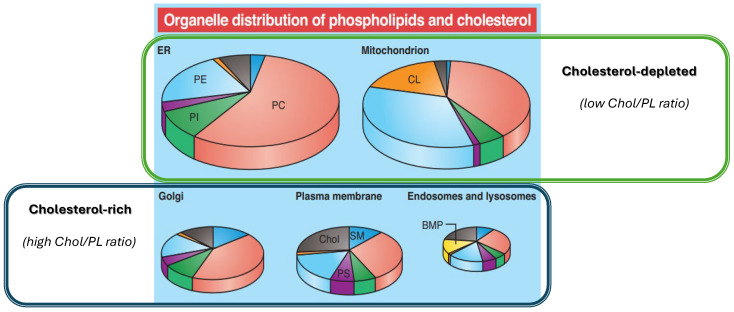
Sub-cellular distribution of main phospholipid classes and cholesterol in plasma membrane and other cell organelles. (Red) PC: Phosphatidylcholine; (light blue) PE: Phosphatidylethanolamine; (purple) PS: Phosphatidylserine; (green) PI: Phosphatidylinositol; (dark blue) SM: sphingomyelin; (yellow) BMP: bis(monoacylglycero)phosphate; (orange) CL: cardiolipin; (grey) Chol: cholesterol. The figure is reproduced from Reis and Dias “Oxysterol sulfates in fluids, cells and tissues: how much do we know about their clinical significance, biological relevance and biophysical implications?” in [2] under the terms of Creative Commons Attribution-NonCommercial 4.0. License.

In the case of endoplasmic reticulum (ER), the organelle membrane is predominantly composed of PC lipids and minor contributions of Chol, phosphatidylethanolamine (PE) and phosphatidylinositol (PI) lipids, whereas cell’s plasma membrane, Golgi complex, lysosomes, and endosomes have a more complex composition rich in PL, Chol, and (glyco)sphingolipids (GSL). Based on their Chol/PL ratios, biological membranes can be generally categorised as cholesterol-poor (low Chol/PL ratio) such as endoplasmic reticulum, and in cholesterol-rich membranes (high Chol/PL ratio) such as plasma membrane and Golgi complex (Figure 1). The distinct and unique lipid composition of biological membranes can be tailored in artificial model membranes.

In an attempt to bring a more comprehensive understanding on the effect of CholS on the biophysical properties of biological membranes, this study describes the incorporation of CholS (2, 5 and 10 mol%) in cholesterol-poor artificial membranes aimed to mimic ER membranes, as well as in cholesterol-rich membranes designed to mimic the plasma membrane of epithelial-like cells (e.g., intestinal, endothelial, oral) and characterisation of the biophysical parameters including anisotropy, lipid order, and surface net charge.

## 2. Materials and Methods

### 2.1. Reagents

Lipids 1,2-myristoyl-sn-glycero-3-phosphocholine (DMPC), 1-palmitoyl-2-lineloyl-sn-glycero-3-phosphocholine (PLPC), 1,2 myristoyl-sn-glycero-3-phosphoethanolamine (DMPE), egg sphingomyelin (N-hexadecanoyl-D-erythro-sphingosylphosphorylcholine, SM) and cholesterol sulphate (CholS) were purchased from Avanti Polar Lipids (Birmingham, AL, USA). Cholesterol (Chol) and fluorescent probes, 1 (4 trimethylammoniumphenyl) 6 phenyl 1,3,5 hexatriene-p-toluenesulfonate (TMA-DPH) and Laurdan, were purchased from Sigma Aldrich (Madrid, Spain). Phosphate-buffer saline (PBS, min 99%) tablets were purchased from PanReac AppliChem (Darmstadt, Germany). Chloroform and methanol were HPLC grade and purchased from Fisher Scientific (Spain). Ultrapure deionised water further purified in a Milli Q Reagent Water System apparatus (InterLab, Almada, Portugal) was used and the specific electrical resistance was <18.2 MΩcm^−1^.

### 2.2. Liposome Preparation

Liposomes were formed by the hydrating extrusion method [28]. Briefly, lipids were dissolved in chloroform:methanol (1:1, *v*/*v*) and mixed in a round-bottom pear-shaped glass flask. The organic solution of each lipid mixture (PLPC/Chol or PLPC/Chol/SM/DMPE) was then divided in 4 aliquots of equal volume. To 3 of the 4 aliquots, CholS dissolved in MeOH (stock solution 7.5 mg/mL) was added to the organic lipid mixtures (to correspond to 2%, 5%, and 10% (mol%) thus enabling to increase the content of CholS while keeping the same phospholipid-to-cholesterol ratio (Chol/PL) in the different aliquots for each system (Supplemental Table S1). Aliquots for each system studied (cholesterol-poor and cholesterol-rich) were evaporated to dryness and kept under vacuum for at least 3 h to remove all traces of organic solvent. Multilamellar vesicles (MLVs) were obtained by redispersion of the lipid films in PBS (10 mM, pH 7.4) to a final concentration of approximately 1 mM. To achieve this, the volume of added PBS was corrected in the aliquots containing CholS in order to maintain the same lipid concentration (1 mM). MLVs were processed by interchangeable freeze/thaw cycles (5) in liquid nitrogen and boiling water bath. Liposome suspensions were vortexed and extruded in a LIPEX Biomembranes extruder attached to a CLIFTON thermostatic circulating water bath above the phase transition temperature (75 °C) using polycarbonate filters (100 nm pores, Whatman^®^, Maidstone, UK) to obtain large unilamellar vesicles (LUVs). For anisotropy experiments, fluorescent probe TMA-DPH dissolved in methanol (stock solution 2.84 mM) was added to the lipid organic mixture in a probe:lipid ratio of 1:300 (mol/mol) followed by drying, hydration, and extrusion steps as stated above. For lipid order experiments, fluorescent probe Laurdan dissolved in chloroform (stock solution 2.13 mM) was added to the lipid organic mixture in a probe:lipid ratio of 1:500 (mol/mol) followed by drying, hydration and extrusion steps as stated above. The LUVs were kept protected from light until spectroscopic characterisation.

### 2.3. Fluorescence Measurements

Anisotropy measurements were performed on TMA-DPH fluorescent-labelled liposomes in a Varian Cary Eclipse spectrofluorometer equipped with a temperature cell holder (Peltier Multicell Holder, Cary Temperature Probe series II) coupled to a water-circulation thermostat. For all systems studied, liposomes were placed in 1.5 mL quartz cuvettes (Starna Scientific, Essex, UK) and steady-state fluorescent anisotropy measurements were recorded at 37 °C. The temperature of samples was monitored using an in-house built sensor immersed inside the quartz cuvette. The excitation (λ_exc_) and emission wavelengths (λ_em_) used were 355/430 nm and slit widths were set to 5 nm for both excitation and emission values. Anisotropy values (<*r*>) are defined by Equation (1):

(1) $$<r>=\frac{I_{VV}-I_{VH}G}{I_{VV}+2\;I_{VH}G}$$

where *I_VV_* and *I_VH_* represent the emission intensities when the emission polarizer was oriented vertical (parallel) and horizontal (perpendicular) to the polarisation of the excitation light. G is a correction factor and is given by the ratio of vertical to horizontal components when the excitation light is polarised in the horizontal direction, *G* = *I_HV_*/*I_HH_* [29]. Anisotropy values shown are the mean of three independent measurements (n = 3). Lipid order measurements were conducted on Laurdan labelled liposomes with increasing amounts of CholS (mol%) at 37 °C. Measurements were carried out in a 10 mm quartz cuvette (Starna Sci, Essex, UK) and emission spectra were recorded at an excitation wavelength of 350 nm and emission wavelength from 380 to 600 nm with slits set at 5 nm for both excitation and emission. The generalised polarisation (GP) was estimated using Equation (2) [30]:

(2) $$GP=\frac{I_{440}-I_{490}}{I_{440}+I_{490}}$$

where *I*_440_ and *I*_490_ represents fluorescence intensity at 440 and 490 nm, respectively.

### 2.4. Measurement of Surface Net Charge

The net surface charge of membrane models was calculated using electrophoretic mobility measured in a Malvern Zetasizer PanAnalytical Nano ZS device (Malvern, UK) at 37 °C with light detection at 17° using disposable plastic cells purchased from Malvern Instruments (PanAnalytical, Malvern, UK). Each sample was measured in triplicate, and each measurement was the average of 20 readings. Zeta potential values were calculated from raw data using the Henry equation with the Smoluchowski approximation (Malvern Instruments software, version 7.13).

### 2.5. Dynamic Light Scattering Measurements

The thermotropic behaviour of LUVs was achieved by measurement of mean count rate versus temperature in a Malvern Zetasizer PanAnalytical Nano ZS device (Malvern Instruments, Malvern, UK), as described previously [31]. Measurements were carried out in capped 3.5 mL transparent glass cuvettes (Starna Scientific, Essex, UK) using a helium–neon laser (λ = 633 nm) at an angle of 173° with fixed attenuation (6) over the temperature range from 2 to 80 °C with 2 °C increments. For each system, liposomes were left to equilibrate for 3 min at each set temperature value before mean count rate measurements. The raw mean count rate data were analysed to obtain the first-derivative plot in Origin (version 9.0) and determine the transition temperatures (T_m_) of each system.

### 2.6. Statistical Data Analysis

Statistical analysis was conducted using GraphPadPrism 8 software (v 8.4.3.). The results are presented (in triplicate) as mean ± standard deviation (± SD) and significance was estimated by one-way ANOVA using Bonferroni’s multiple comparison test. Values with *p* < 0.05 were considered statistically significant.

## 3. Results

Investigations were carried out in two membrane models characterised by low Chol/PL ratio and composed of PLPC/Chol (95:5, mol%) and by high Chol/PL ratio composed of PLPC/Chol/SM/DMPE (30:25:30:15, mol%). The models were tailored to mimic the ER membrane composed mostly of PC and Chol and also the plasma membrane, Golgi complex, lysosomes, and endosomes with a more complex composition of PC and PE as the main PL, together with Chol and GSL (depicted in Figure 1) and in this way bringing additional biological relevance to biomimetic models. In the cholesterol-rich model, while DMPE is not a major lipid of the plasma membrane composition, saturated PE is more chemically stable to non-enzymatic glycation reaction than predominant unsaturated PE. The biomimetic models are characterised by distinct lipid ordering effects, as shown by the GP values estimated (Table 1) for cholesterol-poor and cholesterol-rich models using the fluorescence emission spectra of Laurdan-labelled liposomes obtained in the absence of CholS (Supplementary Figure S1).

No phase diagrams are available in the literature for the specific lipid mixtures used in this study despite the many phase diagrams available in the literature for lipid mixtures of membrane “raft” microdomains [32,33] and few other lipid mixtures [34,35]. At physiological temperature, cholesterol-poor binary systems composed of unsaturated PLPC/Chol are predominantly in the liquid disordered (L_d_) phase [34,35]. Despite the inexistence of phase diagrams for quaternary systems, the work by Kaiser and colleagues (2009) on cholesterol-rich POPC/ESM/Chol model (45:30:25, mol%) in close similarity with the cholesterol-rich epithelial-like model here studied shows coexisting *l_o_*/*l_d_* phases [36].

Of note, the lipid composition (mol %) for the membrane models included in this study reflects the average lipid composition and does not take into account the variations within different cells derived from different species [26,27,37], or the asymmetrical phospholipid distribution occurring in organelles (e.g., mitochondria) and plasma membrane [38,39] crucial for protein function [40].

CholS was incorporated to biomimetic models in amounts that varied between 2 and 10 mol%, values based on the levels of CholS described in the literature [7,8] and characterised according to their biophysical properties by spectroscopic techniques.

### 3.1. Effect of Chol Incorporation in Cholesterol-Poor Membrane Model

Cholesterol-poor artificial membranes mimicking ER membranes and composed of PLPC:Chol (95:5, mol%) were incorporated with increasing amounts of CholS (2, 5 and 10 mol%) and characterised by spectroscopic techniques according to their anisotropy (<r>), membrane order (GP) and surface zeta potential (ζ). CholS is a charged molecule located at the aqueous–lipid interface likely to impact the biophysical properties of biomembranes at the polar headgroup region. Hence, the polar TMA-DPH probe anchoring at the lipid/water interface was used in this study.

**Figure 2:**
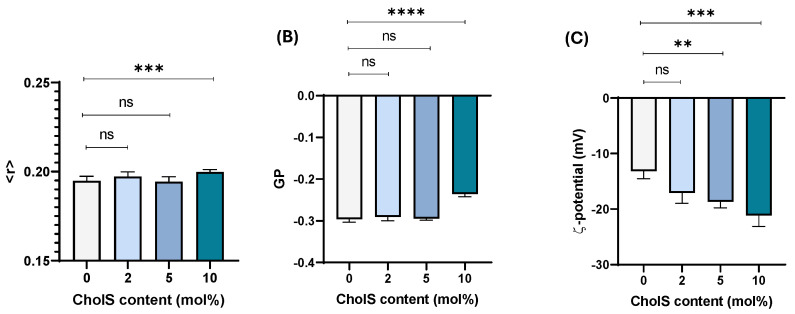
Biophysical characterisation of cholesterol-poor PLPC/Chol model (95:5, mol%) containing different amounts of CholS (0%, 2%, 5%, and 10%, mol%). (**A**) anisotropy (<r>) measurements for TMA-DPH labelled liposomes; (**B**) membrane order parameter in Laurdan-labelled liposomes; and (**C**) zeta-potential (ζ) measurements. All measurements were performed at 37 °C. Data are expressed as mean ± SD (*n* = 3). Statistical significance was ** *p* < 0.005, *** *p* < 0.0005 and **** *p* < 0.0001, ns, not significant.

Results obtained are depicted in Figure 2. Anisotropy measurements conducted at physiological temperature (37 °C) in TMA-DPH labelled PLPC/Chol liposomes show a slight increase in anisotropy (<r>) values for higher CholS amounts (10 mol%) (Figure 2A) reflecting a slight membrane rigidification with the incorporation of CholS. Similarly, only high CholS amounts (10 mol%) induced changes to the lipid order parameter (Figure 2B) in agreement with previous experimental and theoretical studies conducted on single PC membranes [21,24,25]. As anticipated, increasing amounts of the negatively charged CholS resulted in a decrease in surface potential (Figure 2C).

### 3.2. Effect of CholS in Cholesterol-Rich Epithelial-like Membrane Model

To complement findings from cholesterol-poor models, the complex cholesterol-rich model composed of PLPC/Chol/SM/DMPE (30:25:30:15) mimicking the plasma membrane of epithelial-like cells was also studied. Increasing amounts of CholS (2, 5 and 10 mol%) were incorporated in the model and characterised according to its anisotropy (<r>), membrane order (GP) and surface zeta potential (ζ).

**Figure 3:**
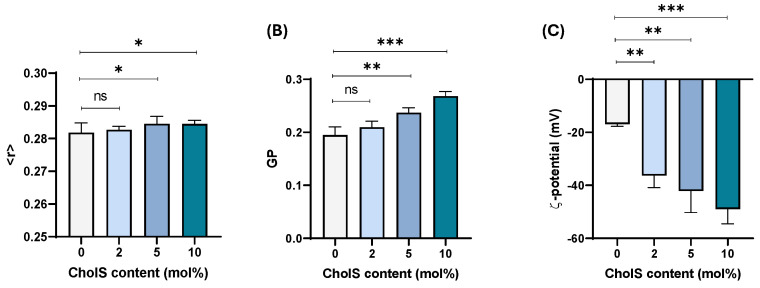
Biophysical characterisation of cholesterol-rich plasma membrane PLPC/Chol/SM/DMPE (30: 25:30:15, mol%) model containing different amounts of CholS (0%, 2%, 5% and 10%). (**A**) anisotropy (<r>) measurements in TMA-DPH labelled liposomes; (**B**) membrane order parameter in Laurdan-labelled liposomes; and (**C**) zeta-potential measurements. All measurements were performed at 37 °C. Data are expressed as mean ± SD (*n* = 3). Statistical significance was ns, not significant, * *p* < 0.05, ** *p* < 0.005, and *** *p* < 0.0005.

Incorporation of increasing amounts of CholS resulted in a minor increase in the <r> values (Figure 3A) suggesting a slight rigidification effect. In addition, fluorescence emission spectra of Laurdan-labelled liposomes at physiological temperature (37 °C) show a slight increase in the membrane order with increasing amounts of CholS (Figure 3B). Similarly to previous cholesterol-poor models, increased CholS content led to the decrease in the surface ζ-potential to more negative values (Figure 3C).

### 3.3. Thermotropic Behaviour of Cholesterol-Poor and Cholesterol-Rich Models

**Figure 4:**
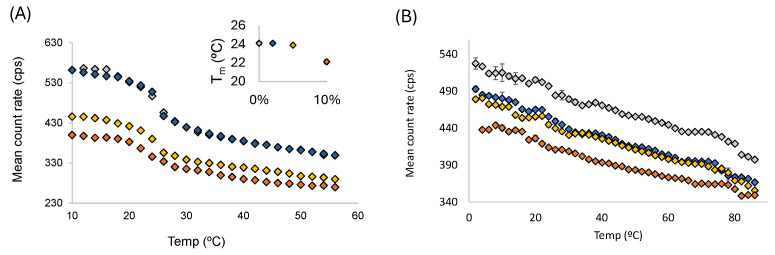
Plot of mean count rate over temperature range for cholesterol-depleted systems (**A**) DMPC/Chol liposomes (95/5) and (**B**) for cholesterol-rich PLPC/Chol/SM/DMPE liposomes (30/25/30/15), with various CholS concentrations (0%: grey diamonds, 2%: blue diamonds, 5%: yellow diamonds, 10%: orange diamonds). Inset depicts the plot of transition temperatures estimated for PLPC/Chol system by first derivative (Origin software) with increasing CholS amounts (0, 2, 5 and 10 mol%).

Previous investigations on the influence of CholS in single DPPC membranes found that the addition of CholS decreased the main transition temperature (T*_m_*) having a more marked fluidising effect than Chol [24]. Because the T*_m_* of PLPC is less than zero, the effect of increasing amounts of CholS on the thermotropic behaviour of PLPC/Chol systems was estimated in the binary model built with DMPC (T*_m_* = 24 °C) with a fluid phase at the physiological temperature [41]. The mean count rate plots obtained as a function of temperature for DMPC/Chol in the absence of CholS (Figure 4A) show a clear gel-to-fluid transition centred at T*_m_* = 24 °C.

Although the incorporation of CholS has no apparent effect on the T*_m_* values, by plotting the first order values obtained for the various amounts of CholS tested there a is noticeable slight decrease in the T*_m_* with the incorporation of high CholS concentrations (inset in Figure 4A) in agreement with previous calorimetry measurements [24]. The mean count rate reflects the changes to the optical properties of the artificial models occurring during phase transitions and detected as differences in the scattering intensity of samples. Mean count rate plots provide a good estimate of the phase transition temperature [31] including binary and ternary systems [42,43], and due to its simplicity it is a major advantage when compared to thermal techniques such as differential scanning calorimetry (DSC) [41,44], and other more expensive and skilled spectroscopic techniques such as ESR [45], atomic force microscopy [46], and nanoplasmonic sensing [47]. The decrease of up to 2.1 °C observed upon the incorporation of 10 mol% of CholS to the DMPC/Chol is in agreement with the 2.4 °C change reported in DPPC liposomes incorporated with 10 mol% determined by DSC [24], suggesting a slight fluidification upon incorporation of CholS. Although the effect of CholS on the thermotropic behaviour of PLPC/Chol cannot be measured, it is likely to induce the same effect and the decrease in the system’s T*_m_*. Contrary to the cholesterol-poor systems, the abolishment of gel-to-fluid transition in the cholesterol-rich plasma membrane model (Figure 4B) reflects the interaction of cholesterol with the surrounding liquid-disordered microdomains formed by unsaturated phospholipids (PLPC) preventing them from packing tightly together. However, in complex systems with coexisting Liquid ordered (L_o_) and Liquid-disordered (L_d_) (micro)domains [36], the role of cholesterol is much more complex as, depending on the lipid composition and temperature, cholesterol can order disordered phases and disrupt gel phases [48].

## 4. Discussion

Our biophysical study on cholesterol-poor and cholesterol-rich biomimetic models shows that the incorporation of CholS impacts the organisation of biomembranes displayed by the small influence on anisotropy, and consequently on membrane fluidity, which is also accompanied by an increased ordering effect (Figure 2A,B and Figure 3A,B). The higher effect of CholS on membrane order, and not on anisotropy, should be interpreted bearing in mind the distinct locations of fluorescent Laurdan (membrane order) and TMA-DPH (anisotropy) probes within the bilayer [49,50]. While TMA-DPH is located within the polar head region and CholS at the water–lipid interface, our findings suggest that CholS may be slightly immersed in the bilayer closer to the carbonyl groups [51] and hence the higher sensitivity observed for Laurdan experiments.

Data from this study also shows that incorporation of CholS strongly impacts the surface potential of both models; though, in this case, the effect seems to be influenced by the lipid composition. Taking into consideration data obtained for cholesterol-poor (Figure 2C) and cholesterol-rich models (Figure 3C), the decrease in the net surface potential was not observed to the same extent in both models even though the same amount of CholS was added (2–10 mol%). As can be seen for the PLPC/Chol system, the surface potential at the highest level of CholS incorporation (10 mol%) was nearly doubled to that exhibited at 0 mol%, whereas the value tripled for the PC/Chol/SM/PE systems. The reasons may be diverse, though we speculate that, much like the distinct location/orientation of cholesterol depending on the hydrophobic thickness [52], the bulky sulphate charged group may adopt distinct location/orientation in more fluid PLPC/Chol membranes when compared to more rigidified PC/Chol/SM/PE membranes, thus exposing the charge to different extent. Though the thermotropic behaviour for PLPC/Chol and complex epithelial-like model (PLPC/Chol/SM/DMPE) could not be obtained, parallel studies conducted on DMPC/Chol systems revealed a fluidification of the system with increasing CholS content. The findings from DMPC/Chol are related to PLPC/Chol as increasing amounts of CholS also led to a slight increase of anisotropy values (10 mol% CholS), increased ordering effect (higher GP values), and a decrease in zeta-potential (to more negative values) (Supplementary Figure S2).

**Figure 5:**
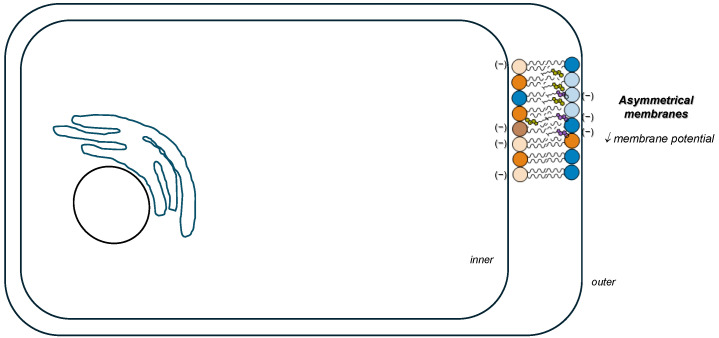
Schematic representation of CholS on the cell membrane transmembrane potential. The presence of surface located CholS (purple) on the outer leaflet in asymmetrical cholesterol-rich membranes (e.g., plasma membrane) composed of PC (blue), PE lipids (orange), sphingomyelins (light blue) and anionic PS (light orange) and PI (brown) lipids, confer additional negative charge decreasing the membrane potential between the two leaflets.

In a broader context, and given the asymmetric (phospho)lipids distribution in the plasma membrane with anionic PL such as phosphatidylserine (PS) and phosphatidylinositol (PI) found almost exclusively in the inner leaflet and zwitterionic PC, SM, and Chol predominantly located to the outer leaflet, the presence of CholS in cholesterol-rich membranes tends to decrease the difference between the transmembrane potential (Figure 5), with an impact on protein–protein interactions and the activity of transmembrane proteins involved in cell adhesion/fusion (endocytosis/exocytosis) and cell signalling events [53].

Curiously, and despite the CholS’ pivotal role in lipid trafficking and in cell–cell mediated signalling events [13,16,17,18,19], this is the first report describing comprehensively the impact of increasing amounts of CholS on the biophysical properties of artificial models mimicking membranes of cell and organelles, thus bringing additional insights to the current knowledge. Several factors may contribute to the scarcity of works and the current lack of interest on the biophysical impact of CholS in biological membranes, namely (1) the poor knowledge on the CholS biological significance, namely its basal levels in cells and tissues; its spatio-temporal distribution in cell membranes and organelles and any changes associated with age, gender, and diet in health and disease [2]; (2) the poor knowledge on the lipidome of epithelial-like plasma membrane and other cell organelles; and (3) the lack of knowledge on the lipid remodelling undergone by biological membranes under pathological conditions. These are crucial details that can be tailored in artificial biomembranes and are currently poorly studied.

Despite this, the interest in the biophysical and biological significance of CholS to biological membranes is expected to shift considering recent works on the microbial biotransformation of CholS [6] and its physiological role in disease [14,20]. Gut Bacteroides phyla microbes living in the human intestine were recently discovered to possess sulfotransferase (SULT) activity and to be responsible for the microbial formation of CholS in the human gut providing an additional pathway for the endogenous formation of CholS [6]. Given the reported role of CholS in preventing exacerbated inflammatory response in colitis animal models and ulcer healing and alleviation of gut inflammation [14,20], the increased ordering effect caused by CholS incorporation in cholesterol-rich epithelial-like membranes may provide the optimal bilayer (micro)environment necessary for ideal protein activity and likely to account for the reported decreased inflammatory response in CholS-treated animal models [14,20]. In fact, previous work found that the presence of CholS in PC bilayers was responsible for the reorientation of matrix metalloproteinase-7 (MMP-7), a membrane protein involved in the inflammatory events of mucosal epithelia [54], resulting in further insertion of the protease into the bilayer, restricting its diffusion and turning the catalytic cleft away from the membrane possibly maximising the accessibility of the active site to pericellular protein substrates [55].

Despite the comprehensive understanding of the biophysical relevance of CholS at the membrane level herein described, there are still some caveats that limit the full scope of our findings. For instance, the lipid composition of biological membranes here included is an average value and does not consider the membrane asymmetry ascribed to plasma membranes and other cellular organelles (e.g., lysosomes) [38]. The average value of some (phospho)lipid classes may be incomplete or unknown. For instance, while the phospholipidome of cholesterol-rich plasma membranes in mammalian epithelial cells (e.g., oral, intestinal, lung, endothelial) is fairly known [56,57,58], very little is known about the full lipidome, namely the cholesterol and GSL content. Preliminary studies have already shown that the cholesterol content in the plasma membranes of epithelial cells may actually reach 40–50 mol% [59,60], a value that greatly surpasses that included in this model and others [36,61,62]. Also, and despite the advances in mass spectrometry-based lipidomics, very little is known about the GSL profile in biomembranes, particularly that of glycosylated sphingolipids such as lactosylceramides (LacCer), sulfatides, gangliosides and globosides, and Forsmann lipids.

The biophysical impact resulting from the incorporation of CholS in both cholesterol-poor and cholesterol-rich plasma membrane models described here provides an investigation currently missing in the literature. The findings here reported gain particular relevance given the recent discovery of microbial pathways in the formation of CholS [6] and the role of microbial CholS on the resolution of host immune and inflammation response in intestinal disorders [14,20]. Because of this and given the emerging roles of plant-based diets as key players in restoring the diversity of microbial ecology and in turn gut eubiosis and functionality [63,64,65], additional studies with cell models mimicking (patho)physiological conditions are needed to corroborate the biophysical findings described here with biomimetic models opening new avenues for the use of nutritional-based strategies in the treatment and management of IBD clinical symptoms in genetically susceptible individuals.

## 5. Conclusions

In this study, our investigations on the impact of physiological levels of CholS on the biophysical properties of cholesterol-poor and cholesterol-rich membrane models show a negligible effect on anisotropy regardless of its lipid composition and cholesterol content. Data also shows that CholS has an overall increasing ordering effect on membrane organisation and on surface potential that is influenced by the lipid composition and cholesterol content. Nonetheless, further work is still needed in order to improve our knowledge on the (patho)physiological levels of CholS in cells and organelles. Once this knowledge is gained and incorporated to tailor more physiologically relevant biomimetic models, it will surely improve our understanding on the role of CholS in membrane-driven events.

## Figures and Tables

**Table 1:** Biophysical parameters measured for cholesterol-poor and cholesterol-rich liposomes in the absence of CholS (0 mol%). Generalised polarisation (GP) values were obtained from the emission spectra Laurdan-labelled liposomes recorded at 37 °C using Equation (2). Anisotropy and zeta-potential values were recorded at 37 °C. Values depict mean ± S.D. (*n* = 3).

Models	Lipid Composition (mol%)	GP Value	Anisotropy (<r>)	Zeta-Potential (mV)
Cholesterol-poor	PLPC/Chol (95:5)	−0.296 ± 0.007	0.19 ± 0.02	−13.2 ± 1.1
Cholesterol-rich	PLPC/Chol/SM/DMPE (30:25:30:15)	0.195 ± 0.013	0.282 ± 0.003	−16.9 ± 0.7

## Data Availability

Raw data are available upon request to the corresponding author.

## Supplementary Materials

The following supporting information can be downloaded at: https://www.mdpi.com/article/10.3390/membranes15060159/s1, Figure S1: Laurdan fluorescence emission spectra for cholesterol-poor (PLPC/Chol, depicted in blue) and cholesterol-rich (PLPC/Chol/SM/DMPE, depicted in orange) systems at 37 °C. The colour gradient depicts increasing amounts of CholS (0–10 mol%); Figure S2: Biophysical characterisation of cholesterol-poor DMPC/Chol model (95:5, mol%) containing different amounts of CholS (0%, 2%, 5%, and 10%, mol%). (A) anisotropy (<r>) measurements for TMA-DPH labelled liposomes; (B) membrane order parameter in Laurdan-labelled liposomes; and (C) zeta-potential (ζ) measurements. All measurements were performed at 37 °C. Data are expressed as mean ± SD (*n* = 3). Statistical significance was ** *p* < 0.005, *** *p* < 0.0005 and **** *p* < 0.0001, ns, not significant. Table S1: Lipid composition of cholesterol-poor and cholesterol-rich liposomes.

## Abbreviations

The following abbreviations are used in this manuscript:

Chol/PL	Cholesterol-to-Phospholipid ratio
SC	Directory of open access journals
STS	Steroid sulfatase
ESM	Egg sphingomyelin
ER	Endoplasmic Reticulum
GP	Generalised Polarisation
GSL	GlycoSphingoLipid
PC	Phosphatidylcholine lipids
PE	Phosphatidylethanolamine lipids
PL	Phospholipid
RLXI	Recessive X-linked ichthyosis disorder
