# Supplementary material for: Cholesterol Sulfate in Biological Membranes: A Biophysical Study in Cholesterol-Poor and Cholesterol-Rich Biomimetic Models
Source: Membranes (Basel). 2025 May 24;15(6):159. doi: 10.3390/membranes15060159 (PMC12194945; doi:10.3390/membranes15060159)
Supplement: Supplementary file 1 [file membranes-15-00159-s001.zip › membranes-3608917-supplementary/Supplemental Table S1.pdf]

Supplemental Table S1. Lipid composition of cholesterol-poor and cholesterol-rich liposomes.

	Model composition (mol%)				
Cholesterol-poor systems	**PLPC**	**Chol**	**CholS**		
	95	5	-		
	93	4.9	2		
	90.3	4.8	5		
	86.5	4.5	10		
Cholesterol-rich systems	**PLPC**	**Chol**	**SM**	**DMPE**	**CholS**
	30	25	30	15	-
	29.4	24.5	29.4	14.7	2
	28.5	23.8	28.5	14.3	5
	27	22.5	27	13.5	10
