# Supplementary figures and images for: Cholesterol Sulfate in Biological Membranes: A Biophysical Study in Cholesterol-Poor and Cholesterol-Rich Biomimetic Models
Source: Membranes (Basel). 2025 May 24;15(6):159. doi: 10.3390/membranes15060159 (PMC12194945; doi:10.3390/membranes15060159)

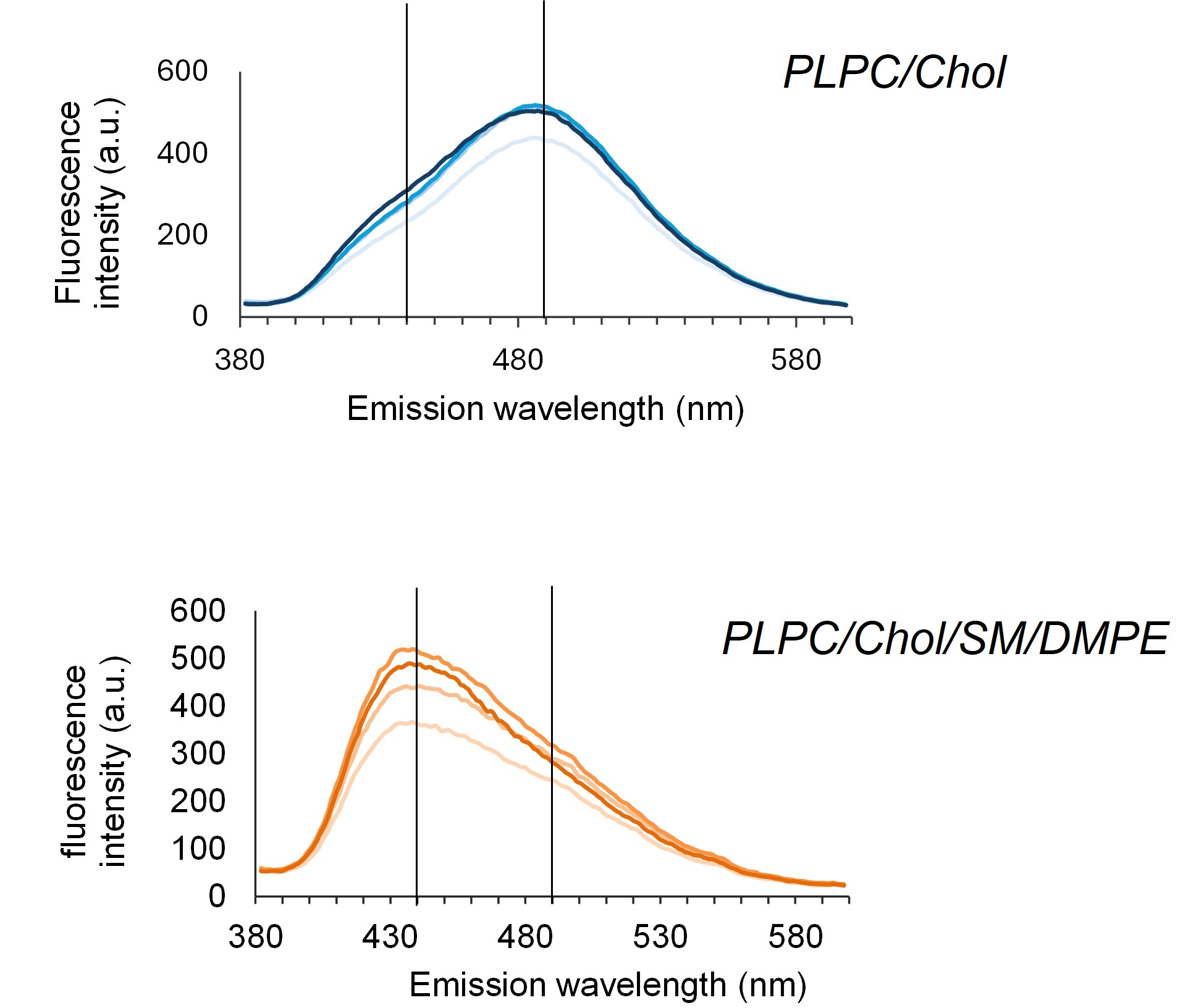

Supplement: Supplementary file 1 [file membranes-15-00159-s001.zip › membranes-3608917-supplementary/Supplemental Figure S1.jpg]

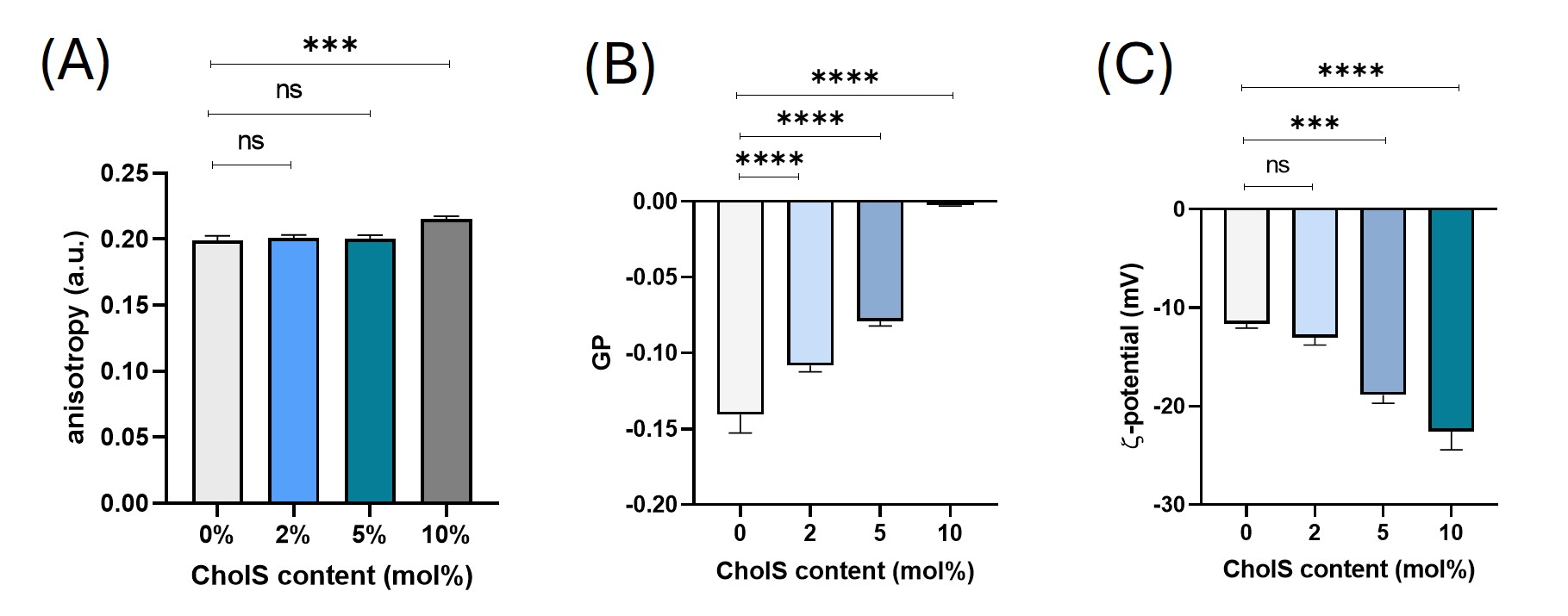

Supplement: Supplementary file 1 [file membranes-15-00159-s001.zip › membranes-3608917-supplementary/Supplemental Figure S2.jpg]
